# Purification of Wastewater from Biomass-Derived Syngas Scrubber Using Biochar and Activated Carbons

**DOI:** 10.3390/ijerph18084247

**Published:** 2021-04-16

**Authors:** Enrico Catizzone, Corradino Sposato, Assunta Romanelli, Donatella Barisano, Giacinto Cornacchia, Luigi Marsico, Daniela Cozza, Massimo Migliori

**Affiliations:** 1 ENEA-Italian National Agency for New Technologies, Energy and Sustainable Economic Development, Trisaia Research Center, Department of Energy Technologies and Renewable Sources, I-75026 Rotondella, Italy; corradino.sposato@enea.it (C.S.); assunta.romanelli@enea.it (A.R.); donatella.barisano@enea.it (D.B.); giacinto.cornacchia@enea.it (G.C.); 2 Department of Environmental and Chemical Engineering, University of Calabria, via P. Bucci, 44a, I-87036 Rende, Italy; luigi.marsico92@gmail.com (L.M.); daniela.cozza@unical.it (D.C.); massimo.migliori@unical.it (M.M.)

**Keywords:** biomass, syngas scrubber wastewater, environmental pollution, pollutant abatement technologies, biochar, adsorption

## Abstract

Phenol is a major component in the scrubber wastewater used for syngas purification in biomass-based gasification plants. Adsorption is a common strategy for wastewater purification, and carbon materials, such as activated carbons and biochar, may be used for its remediation. In this work, we compare the adsorption behavior towards phenol of two biochar samples, produced by pyrolysis and gasification of lignocellulose biomass, with two commercial activated carbons. Obtained data were also used to assess the effect of textural properties (i.e., surface area) on phenol removal. Continuous tests in lab-scale columns were also carried out and the obtained data were processed with literature models in order to obtain design parameters for scale-up. Results clearly indicate the superiority of activated carbons due to the higher pore volume, although biomass-derived char may be more suitable from an economic and environmental point of view. The phenol adsorption capacity increases from about 65 m/g for gasification biochar to about 270 mg/g for the commercial activated carbon. Correspondingly, service time of commercial activated carbons was found to be about six times higher than that of gasification biochar. Finally, results indicate that phenol may be used as a model for characterizing the adsorption capacity of the investigated carbon materials, but in the case of real waste water the carbon usage rate should be considered at least 1.5 times higher than that calculated for phenol.

## 1. Introduction

Phenolic compounds attract great attention in the international scientific community because they are chemicals that are capable of persisting in the environment for long periods of time and can exert toxic effects on humans and animals [1]. Moreover, phenols cause negative effects on drinking water and environment. Some phenolic compounds are abundant in nature and are associated with the colors of flowers and fruits. Others are synthesized and they are the basic ingredient for many synthetic organic compounds, but the high toxicity may affect aquatic life, causing ecological imbalance. Phenol can be absorbed by the human body through the respiratory organs, skin and the alimentary canal [2].

Wastewaters containing phenol may be produced in biomass-based industry [3]. Biomass gasification is the thermochemical process used to convert solid biomass into syngas, a gaseous mixture consisting of hydrogen, carbon monoxide and carbon dioxide, which can find different uses for energy purposes [4,5]. One of the main issues related to biomass gasification is the presence of tars, a group of organic compounds [6] whose presence could make this technology unsuccessful from a commercial point of view [7]. In order to overcome this problem, two strategies may be adopted, namely the optimization of gasification operation conditions with addition of sorbents and catalysts directly in the gasification reactor (primary methods) or syngas cleaning, by means of systems and processes implemented downstream of the gasifier (secondary methods) [8,9,10]. In this regard, several technologies have been developed with the aim to push towards an efficient and economic/environmental sustainable process [11]. Tar is a complex mixture of condensable hydrocarbons, including oxygenated or polycyclic aromatics. Among the several cleaning technologies proposed, tar removal through physical processes are the most used at demonstrative scale. Physical processes may be dry or wet. Adsorbents, activated carbons and catalytic filters may be used as dry technologies for tar removal. On the contrary, the absorption tower (scrubber) is the most used wet technology. The advantage of a scrubber unit is related to the possibility to use the same unit as both quencher and absorber. For instance, after the gasifier, a wet scrubber with an organic liquid (e.g., bio-diesel) may be used for quenching the hot syngas and for removing the hydrophobic and heavy tar molecules from the syngas [12]. The exhausted bio-diesel could be used as fuel for energy production [13].

Afterwards, the content of hydrophilic or light tars, such as phenol, can be further reduced by a secondary wet scrubber using water as absorbent liquid. In that case, the produced wastewater has to be properly treated and phenol is usually the main organic tar to be removed [14].

The concentration of phenolic compounds in scrubber wastewater strongly depend on both phenol content in the syngas and process scheme and parameters, e.g., water-to-syngas flowrate ratio in the wet scrubber unit. For instance, Akhlas et al. report that phenol concentration may be in the range 950–4630 mg/L [15], as a function of the process conditions of the biomass gasification plant, although the lower value, i.e., 772 mg/L, is reported by Panchratna et al. [16]. Lower phenol concentration may be found in the case of coal gasification. In particular, Li et al. [17] report values lower than 500 mg/L for a full scale wastewater treatment facility of a coal gasification plant installed in Harbin (China). A phenol concentration up to 1600 mg/L is reported by Wang et al. for a Lurgi coal gasification wastewater, indicating that phenol concentration in scrubber wastewater strongly depends on the characteristics of the plant also for the coal gasification process [18].

In order to effectively eliminate phenolic compounds from wastewater, different processes are commonly used such as photocatalytic degradation, ozonation, extraction (liquid-liquid or solid phase extraction), biological methods (via microbial or enzymatic methods), membrane-based separation methods, and adsorption [19]. Activated carbons are versatile adsorbents, particularly effective in the adsorption of organic and inorganic pollutants from aqueous solutions [20]. In chemical industry, the most common applications of activated carbon are in fixed bed reactors or fluidized bed reactors in which the passage of a gas or liquid stream permit the removal of a large number of contaminants. Carbon characteristics, such as the large percentage of micropores, high specific surface area, high pore volumes, good mechanical strength, etc., provide a framework type with chemical and physical characteristics useful for absorption application [21]. Activated carbon is commonly used in powder and granular form. Commercially, both forms are sold and the most appropriate is chosen according to industrial applications; other forms such as felts, fibers and cloths are being studied in the scientific research [22].

In the literature, many works have been done on the aromatic compounds’ absorption in aqueous solution and, in particular, phenols attract great attention [23,24,25,26,27,28].

Mukherjee et al. worked on phenol adsorption efficiency of different adsorbents including bagasse ash, activated carbon and charcoal from wastewater [29]. The monitored parameters for evaluating adsorption efficiency was the influence of pH, concentration of ethylenediaminetetraacetic acid (EDTA), anions and adsorbent dose. Their result showed 98, 90 and 90% phenol removal efficiencies by activated carbon, wood charcoal and bagasse ash systems, respectively. Removal efficiency was observed to increase with a decrease in the pH of the system. Effects of EDTA and nitrate ion content of the solution were identified as the factors that influenced the adsorption process.

Akl et al. [30] used sugarcane bagasse-based activated carbons to remove phenol form aqueous solution. They found that the proposed adsorbents are capable of phenol elimination from water. In particular, the process depends on pollutant concentration, pH solution and temperature.

Alhamed [31] studied the kinetics of phenol adsorption on activated carbon produced from waste dates’ stones using four different solid particle sizes (1.47 to 0.225 mm) and initial concentrations of phenol of 200 and 400 ppm. He found that the initial rate of adsorption, predicted from the pseudo-second order model, decreased with increasing particle diameter as a result of higher interfacial area provided by particles with smaller diameter. Breakthrough curves for phenol removal using a packed bed of activated carbon were well predicted using an axial dispersion model.

Daifullah et al. [32] studied the removal of phenol and its derivate by adsorption on activated carbons prepared from H_3_PO_4_-impregnated powdered apricot stone shells and carbonized at 300–500 °C. Uptake of phenols increases with the respective increase in molecular dimensions and acidity of the organic compound and decrease in solubility of the sorbates.

In this work, the phenol removal from aqueous media was carried on by using residual biochars. For comparison of the treatment effectiveness, commercial activated carbons were included. The latter were kindly provided by Sicav S.p.A., both in granular and powder form; these materials were considered as representative of high performance materials with elevated BET specific surface area and pore volume. The considered biochars were agroindustrial biomass residues produced in pyrolysis and gasification processes of concern at the ENEA—Trisaia Research Centre (south of Italy). In this context, in the specific plant configurations, sections for gas cleaning based on combined biodiesel and water scrubbing are under consideration. In fact, the effect of carbon amount on treatment performances of real scrubber water was also assessed.

Therefore, the obtained data were analyzed to evaluate the performance of the experimental biochars in wastewater treatment for possible reuse options of the two residues, with a view to closing the production cycle of the thermochemical processes involved, reducing wastewater and solid process residues to limit the environmental impact and increase the sustainability of the processes.

## 2. Materials and Methods

### 2.1. Materials 

Two activated carbons with specific surface areas of 800 m^2^/g (SP800) and 1000 m^2^/g (SP1000) were kindly provided by Sicav S.p.A (Gissi, Italy) in powder form with sizes < 75 µm and grain form with sizes of 1–1.5 mm. Both samples were used as-received with no additional treatments. The two biomass-derived carbons (biochar) were residual materials produced during processes of pyrolysis (SPBCP) or gasification (SPBCG). The SPBCP sample originated from wood chips subjected to slow pyrolysis carried out at 550 °C for 16 h, while the SPBCG came from a biomass gasification plant. The biomass gasification plant consists of a 120-kWth downdraft gasifier utilizing air as the gasification agent. The produced syngas is then purified in a cleaning section which consists of four units: cyclones, wet scrubbers, demisters and a chips filter. Wet scrubbers consist of two units in series. Heavy tars (mainly hydrophobic molecules) are removed in the primary biodiesel scrubber. Residual light tars, mainly phenolic molecules, are then removed in the secondary water scrubber. In that last unit, wastewater is produced and requires purification. Almond shells were used as feedstock and the gasifier was operated at a temperature of about 850 °C.

Before use, both the biochar samples were ground in a blade mill and then sieved; the powder fraction with size < 75 µm was used for the adsorption tests. To facilitate the grinding, amounts of the two samples were first kept at 70 °C for three days to reduce the moisture content. In order to carry out continuous tests in the column, the produced biochars were also sieved in the range 1–1.5 mm.

### 2.2. Characterization of the Investigated Carbons

All the investigated carbons were analyzed by thermogravimetric analysis in the range 30–850 °C, in air flow, and with a heating rate of 10 °C/min (SDT 650, TA Instruments, New Castle, DE, USA). The produced biochars (namely SPBCP and SPBCG) were also characterized in terms of textural properties. In this regard, the specific surface area (SSA) of the produced samples were obtained by adopting the Brunauer–Emmett–Teller model (B.E.T.) to nitrogen adsorption isotherms performed at 77 K with an ASAP 2020 Micromeritics instrument. In particular, the B.E.T. was applied in the P/P° range 0.01–0.1 in order to obtain a positive C-value, which is exponentially related to the adsorption energy of the monolayer [33]. Total pore volume was calculated at P/P° = 0.99. Pore size distribution was determined by Non-Local Density Functional Theory (NLDFT) by using MicroActive Version 4.06 software (Micromeritics, Norcross, GA, USA) and by adopting an available model for carbon slit-shape pores. Before the analysis, the investigated samples were degassed, by heating at 300 °C, at a rate of 5 °C min^−1^, under a high vacuum (<10^−8^ mbar) for 12 h. Scanning electron microscope (SEM) images of the investigated samples were collected by a scanning electron microscope (FEI model Inspect, ThermoFisher Scientific, Hillsboro, OR, USA).

### 2.3. Batch Adsorption Tests

The batch phenol adsorption tests were carried out in a glass laboratory bottle using 50 mL of an aqueous solution containing 5 g/L of phenol (PhOH). The mass of adsorbent (activated carbons or biochars) was varied between 0.5 g and 4 g. The adsorption tests were carried out under vigorous stirring at 25 °C and the effect of contact time was investigated in the range 0–24 h. Details are reported in the preliminary study [1].

Batch adsorption tests were also carried out by using scrubber wastewater produced in the demonstrative biomass gasification plant described above. 

After the adsorption test, the solid was separated from the liquid by centrifugation at 4000 rpm for 10 min. Any carbon residues suspended in the liquid were carefully eliminated by vacuum filtration followed by a filtration step through a 0.45 PTFE filter before the HPLC-UV-Vis analysis. UV-Vis analysis was performed by an Agilent 1100 HPLC system coupled with a DAD detector (Eluent: H20/ACN 80/20, supplied in isocratic mode; flow: 1 mL/min; column: C18; λ: 254 nm). The amount of adsorbed phenol as a function of time was fitted with two adsorption kinetic models, i.e., the pseudo-first and -second order models [34], while equilibrium values were modelled with Langmuir, Freundlich and Temkin equations [34,35]. Scrubber wastewater treatment was assessed by analyzing the treated samples with a Varian Cary 300 Scan UV-Vis spectrophotometer (Agilent Technologies, Inc., Santa Clara, CA, USA) in the wavelength range 190–400 nm.

### 2.4. Continuous Adsorption Tests

**Figure 1:**
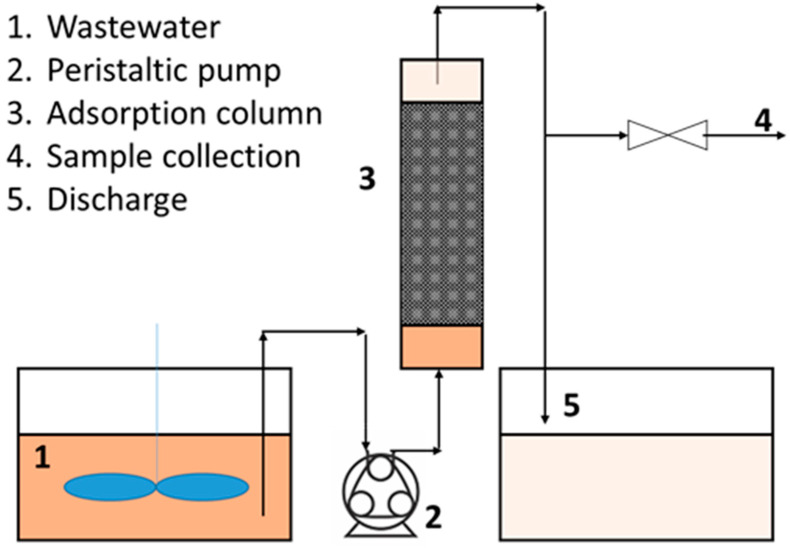
Scheme of the experimental set-up used for continuous adsorption tests.

Continuous adsorption tests were carried out in the lab-scale experimental set-up reported in Figure 1.

The system consisted of a calibrated peristaltic pump that fed the phenol solution to the bottom of a glass cylindrical column containing the carbon.

The column had an internal diameter of 29 mm and a total length of 60 cm. The liquid flowrate was set to 55 mL/min to obtain an empty bed liquid velocity equal to 5 m/h. Different amounts of carbons (namely 20, 30 and 50 g) were used to study the effect of the length of the adsorption bed on the breakthrough curve. Before each test, the carbon bed was purged with water in order to eliminate air contained inside the pores. Furthermore, this purge also allowed the elutriation of the residual fine particulate from the carbon grains. From the top of the column the out-stream was sampled every one minute. The recovered samples were stored at 4 °C in closed bottles and analyzed by the HPLC technique as previously described.

The obtained breakthrough curves were modelled with the Thomas model [36]:

(1) $$\frac{C_{t}}{C_{0}}=\frac{1}{1+exp\left(\frac{k_{Th}}{Q}(q_{Th}m-C_{0}Qt\right)}$$

where *C_t_* is the phenol concentration (unit: mg/mL) in the column outlet at the time *t* (unit: min), *C*_0_ is the initial phenol concentration (unit: mg/mL) in the feed, *k_Th_* is the Thomas constant (unit: mL/min/mg), *q_Th_* is the maximum adsorption capacity (unit: mg/g), *Q* is the wastewater flowrate (unit: mL/min) and *m* is amount of carbon used for the experiment (unit: mg). The Thomas model assumes Langmuir isotherms for equilibrium and pseudo-second-order reaction kinetics [36]. These assumptions agree with the equilibrium and kinetics insights found for the investigated process, as reported in a previous study [1]. One of the main weaknesses of the Thomas model is that its derivation assumes that the adsorption process is limited by kinetics, while it is often controlled by interphase mass transfer. Nevertheless, Thomas is widely used to describe the adsorption process in packed bed columns [37,38,39,40,41].

Furthermore, experimental data were also modelled with the Modified Dose Response (MDR) model [42]:

(2) $$\frac{C_{t}}{C_{0}}=1-\frac{1}{1+{\left(\frac{Q\cdot C_{0}\cdot t}{q_{MDR}\cdot M}\right)}^{a}}$$

where *C_t_* is the phenol concentration (unit: mg/mL) in the column outlet at the time *t* (unit: min), *C*_0_ is the initial phenol concentration (unit: mg/mL) in the feed, *q_MDR_* is the maximum adsorption capacity (unit: mg/g), “*a*” is a MDR parameter (unit: none), *Q* is the wastewater flowrate (unit: mL/min) and M is amount of carbon used for the experiment (unit: g). MDR is an empirical model usually adopted to minimize the error that results from use of the Thomas model, especially with lower and higher breakthrough curve times.

The obtained data were also used for scaling up by adopting the Bed Depth Service Time (BDST) approach [43].

## 3. Results

### 3.1. Characterization of the Samples

**Figure 2:**
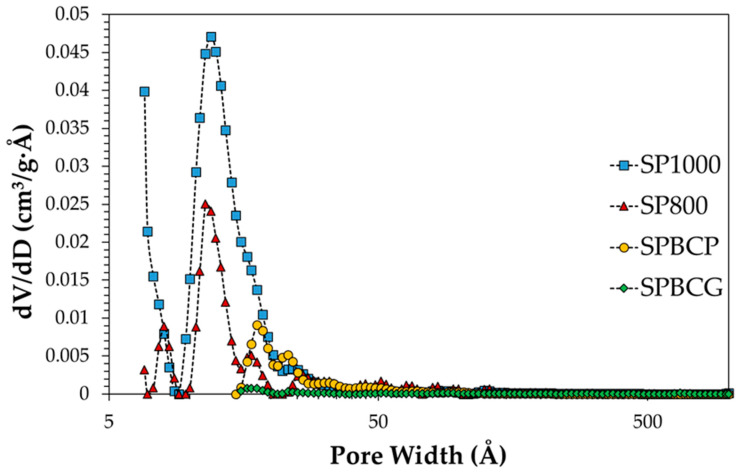
Pore size distribution of the investigated samples.

The B.E.T specific surface area (SSA), B.E.T. C-value, and total pore volume of the investigated samples are reported in Table 1, while pore size distribution is reported in Figure 2. In the literature it is known that, in the case of biomass belonging to similar types, the pyrolysis biochar has a typically specific surface area lower than biochar from gasification. It is also known that the characteristics of the char produced are also strongly influenced by the type of biomass and by the specific morphology [44,45]. These aspects apply well to the case of the chars used in this work, which in fact come from different biomasses, wood chips and almond shells, respectively known for having different textural characteristics (higher porosity in the former, low in the latter) [46,47,48]. Pore size distribution reveals that commercial activated carbons possess pores with an average pore width of about 11–12 Å (Å is Ångström, 10^−10^ m). Smaller pores are not excluded. On the contrary, lager pores, i.e., >15 Å can be observed for the investigated biochar. The pores are suitable for the inclusion of phenol, which has a molecular diameter of 7.46 Å [49].

Thermogravimetry analysis carried out in air flow revealed that the combustion of both the activated carbons occurs in the temperature range 400–600 °C with a maximum heat flow at about 580 °C, indicating a similar structure. On the contrary, the combustion of the biomass-derived chars start at about 250 °C with a maximum heat flow in the range 400–500 °C. After complete combustion, the residual matter was about 5% for SP1000, about 18% for SP800 and about 20% for SPBCG, while it was below 1% for SPBCP. Such differences may be related to both the thermal preparation process and the original feedstock that affects the carbon content and inorganic matter in the produced solid.

**Figure 3:**
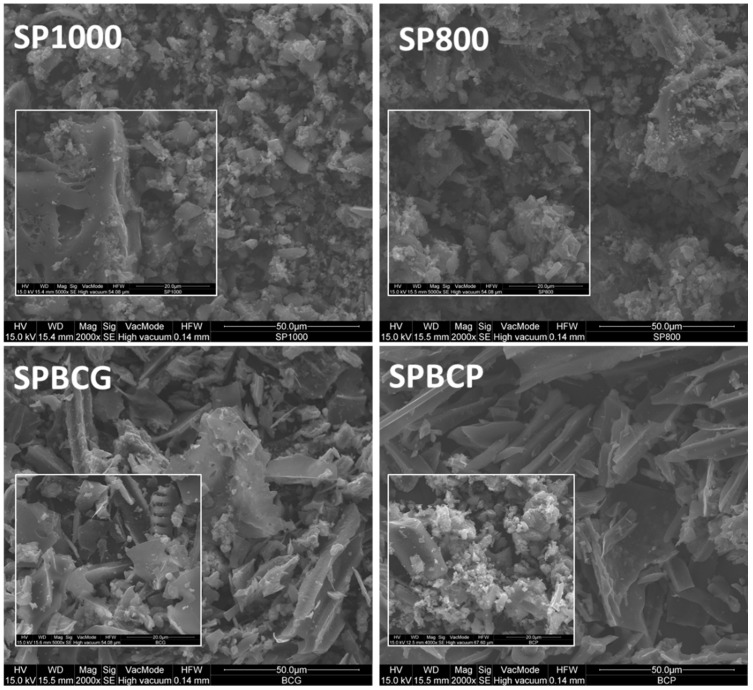
SEM images of the investigated SP1000, SP800, SPBCG and SPBCP samples. Subfigures are SEM images at higher magnification.

Figure 3 shows the SEM images of the investigated samples at two different magnifications. All the samples exhibit a not well-defined shape with macropores that may be observed for the SP1000 sample. The presence of some large pores on the external surface may be also observed for the SPBCG sample. On the contrary, no large pores are observed on the SPBCP sample probably due to the milder conditions adopted for its production (i.e., slow pyrolysis), with respect to the SPBCG sample which was produced in more severe conditions (i.e., high temperature gasification).

### 3.2. Adsorption Tests under Batch Conditions

Batch tests for phenol removal were described and discussed in a previous work [1]. Briefly, the trend of phenol adsorption as a function of time was modelled with both pseudo-first and pseudo-second order kinetics, and F-tests indicate the latter as the best fitting model. In particular, the model also indicates that the SP1000 sample shows an adsorption rate four times higher than the SP800 sample, which displays a kinetic constant one order of magnitude higher than biochars. Concerning biochars, a higher kinetic constant is calculated for the SPBCG sample, despite a lower equilibrium capacity.

The Langmuir model was found to be the best fitting model for equilibrium data. Details about F-tests and comparison with the other models, i.e., Temkin and Freundlich, are reported elsewhere [1]. The Langmuir model predicted a phenol maximum quantity adsorbed at 25 °C equal, respectively, to 270 mg/g, 233 mg/g, 104 mg/g and 65 mg/g, for SP1000, SP800, SPBCP and SPBCG, while the equilibrium constants were, respectively, 1.7 × 10^−2^ L/mg, 3.0 × 10^−3^ L/mg, 5.4 × 10^−4^ L/mg and 9.4 × 10^−4^ L/mg.

**Figure 4:**
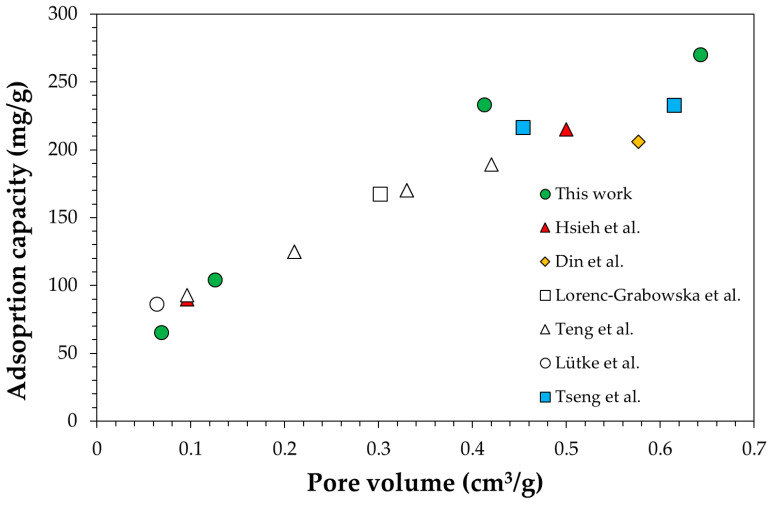
Langmuir adsorption capacity at 25 °C as a function of pore volume. Comparison with literature data [50,51,52,53,54,55].

In particular, the calculated adsorption capacity increases as a function of pore volume as reported in Figure 4, also according to literature data [50,51,52,53,54,55]. In contrast, no trend was found for the Langmuir equilibrium constants. In fact, phenol adsorption appears to be more favorable on SPBCG than on SPBCP, despite the smaller surface area, indicating that the investigated biochars exhibit different phenol–surface interactions.

Obtained data indicate SP1000 and SPBCP as the best carbons among the investigated activated carbons and biochars, respectively. Nevertheless, the utilization of the SPBCG sample for wastewater treatment may be interesting from a process circularity perspective. In fact, the char produced during the biomass gasification process is considered as a low value material, with very limited uses, to be disposed of as a process waste stream. Therefore, the utilization of biomass gasification char as adsorbent material for the treatment of wastewater produced during syngas wet cleaning may be an interesting strategy for its valorization. Biochar from gasification may be considered as a zero-value product (that may become a negative value in the case it is disposed of as waste), while biochar from pyrolysis usually has a positive production cost, since it is a target product of the process. Therefore, albeit that the SPBCG sample exhibits the lowest capacity of adsorption towards phenol, it was studied as a potential adsorbent for the treatment of real scrubber wastewater produced in a biomass-based gasification plant. For comparison, the SP1000 sample was also studied in terms of capacity of purification of real syngas scrubber wastewater.

**Figure 5:**
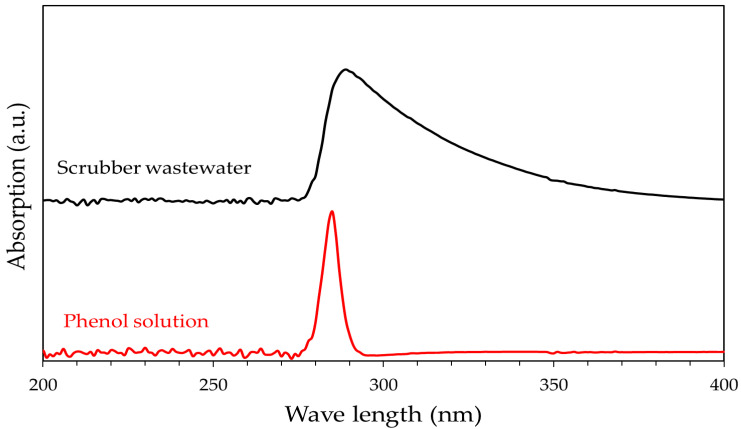
UV-Vis profiles of real syngas scrubber wastewater and 5 g/L phenol solution used in this work.

UV-Vis profiles of the real syngas scrubber wastewater and phenol solution used in this study are reported in Figure 5.

UV-Vis spectra of the phenol solution exhibit a well-defined absorption band centered at about 285 nm. On the contrary, a broad band is observed for the syngas scrubber wastewater indicating the presence of several compounds. However, also for the investigated wastewater the maximum absorption occurs at about 285 nm, suggesting that phenol may be considered a molecule suitable for modelling.

**Figure 6:**
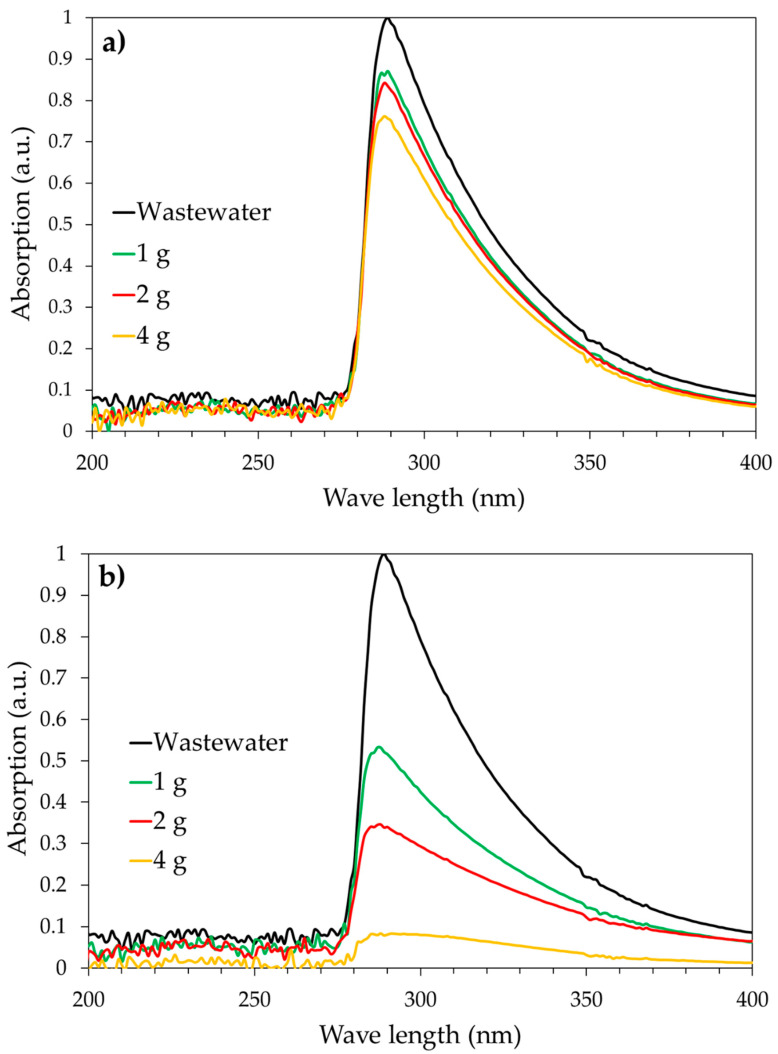
UV-Vis profiles of scrubber wastewater as a function of carbon amount (50 mL of solution for each test) for the SPBCG sample (**a**) and the SP1000 sample (**b**).

**Figure 7:**
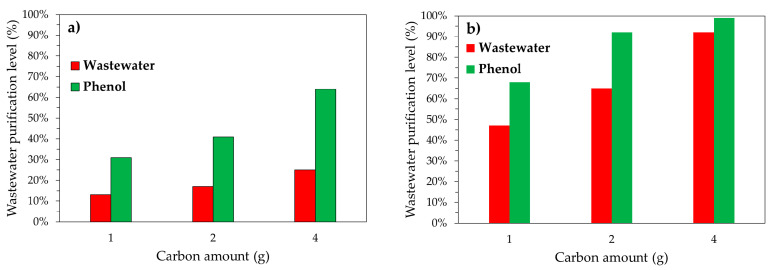
Scrubber wastewater and phenol model solution purification level as a function of carbon amount for the SPBCG (**a**) and SP1000 (**b**) samples, after 24 h treatment.

The UV-Vis spectra of the syngas scrubber water as a function of carbon amount after 24 h of treatment are reported in Figure 6. The effect of carbon amount of wastewater treatment level is reported in Figure 7.

Results clearly confirm the superiority of activated carbons over biochar in terms of purification level for both scrubber wastewater and phenol removal.

The purification level of both real scrubber wastewater and phenol model solution increases as the carbon amount increases.

In particular, the purification level of wastewater and phenol solution increases from 13% to 25% and from 31% to 64%, respectively, by increasing the SPBCG amount from 1 g to 4 g. In the case of commercial activated carbon SP1000, the purification level increases from 47% to 92% in the case of real syngas scrubber wastewater, and from 68% to 99% in the case of phenol model solution.

In fact, as an example, with 4 g of carbon, a wastewater purification level of about 92% is achieved for commercial activated carbon SP1000, while only 25% is measured for gasification biochar SPBCG. A similar trend is also observed in terms of phenol removal.

It is important to observe that the purification level is higher in the case of phenol model solution. As an example, with 4 g of carbon, the purification level was 64% for phenol model solution and 25% for scrubber wastewater in the case of SPBCG, and 92% and 96% for phenol model solution and scrubber wastewater, respectively, in the case of commercial activated carbon. This experimental evidence is of paramount importance. In fact, phenol is usually considered as a model for such a kind of investigation but the complexity of the real wastewater stream may have a significant impact on adsorption performances.

### 3.3. Adsorption Tests in Continuous Mode: Experimental, Modelling and Scale-Up

Continuous tests at bench-scale were carried out over the SP1000 and SPBCG samples, available in the size range 1–1.5 mm. As previously observed, SP1000 is the sample with the best adsorption properties in terms of both kinetics and thermodynamics. On the contrary, SPBCG showed the slower adsorption kinetics and the lower adsorption capacity. However, the potential utilization of SPBCG may be also related to economic consideration. For instance, phenol-contaminated wastewater may be produced in biomass-fueled gasification plants. To regenerate a such waste stream, it may be both environmentally and economically beneficial to consider an on-site treatment with the biochar produced as a residue of gasification rather than its disposal.

**Figure 8:**
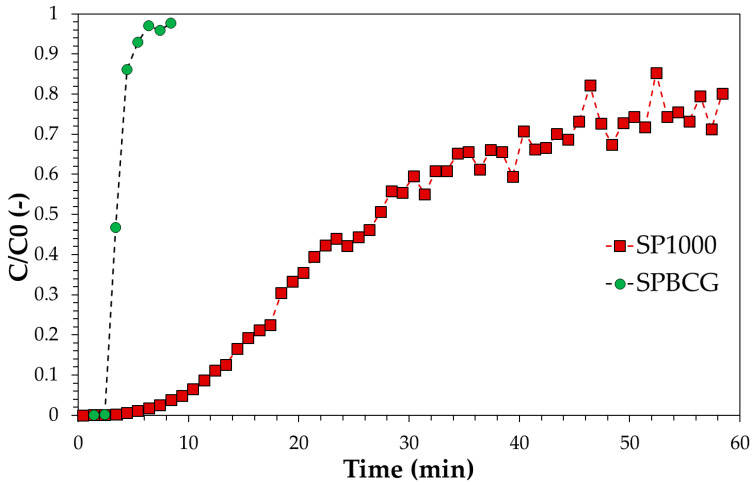
Breakthrough curve of the SP1000 and SPBCG samples for phenol model solution. Solution initial concentration: 5000 mg/g, flowrate: 55 mL/min, carbon amount: 50 g.

The breakthrough curves comparing the adsorption activity of commercial activated carbon vs. the investigated gasification biochar are reported in Figure 8. Assuming a C/C0 value at the breakpoint equal to 0.1, under the conditions adopted the collected data allow estimation of service times of approximately 12 min for the SP1000 sample and 2–3 min for the SPBCG sample. Therefore, with the same fluid dynamic conditions, pollutant load, and service time, these outputs indicate that the quantity of biochar required is at least four times the quantity of commercial activated carbon. This difference may increase for effluents with lower phenol concentrations, due to the differences observed in the adsorption isotherms. In particular, commercial activated carbon exhibits an almost irreversible isotherm, while gasification biochar shows a much less favorable adsorption, with a less pronounced increase in the quantity adsorbed with the solution concentration.

**Figure 9:**
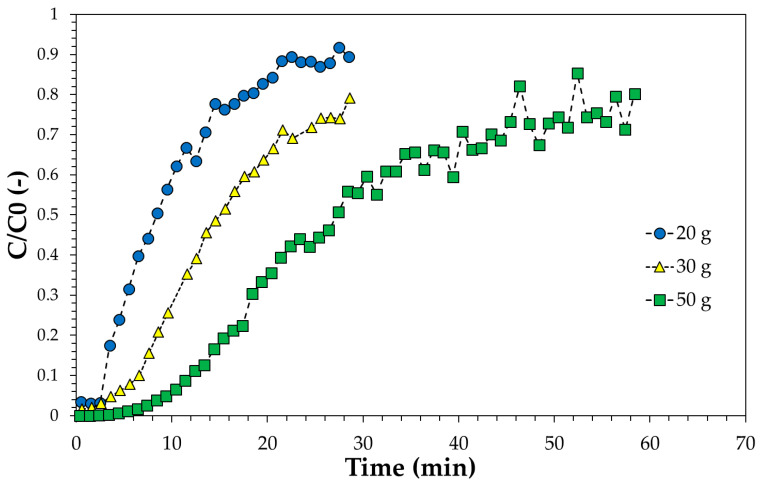
Breakthrough curve of SP1000 as a function of carbon amount for the phenol model solution. Solution initial concentration: 5000 mg/g, flowrate: 55 mL/min.

The effect of adsorption bed weight on breakthrough curve was studied for the SP1000 sample and results are reported in Figure 9.

From the mass balance it is possible to calculate the characteristic parameters reported in Table 2.

The reported data show how the abatement level increases as the height of the bed increases. The total adsorbed quantity increases from 143 to 177 mg/g, demonstrating that column is not operating in equilibrium conditions, the equilibrium adsorption capacity being equal to about 270 mg/g. As the height of the bed increases, the phenol adsorption capacity at the breakpoint also increases.

As previously reported, the obtained breakthrough curves were modelled with both the Thomas and MDR models.

**Figure 10:**
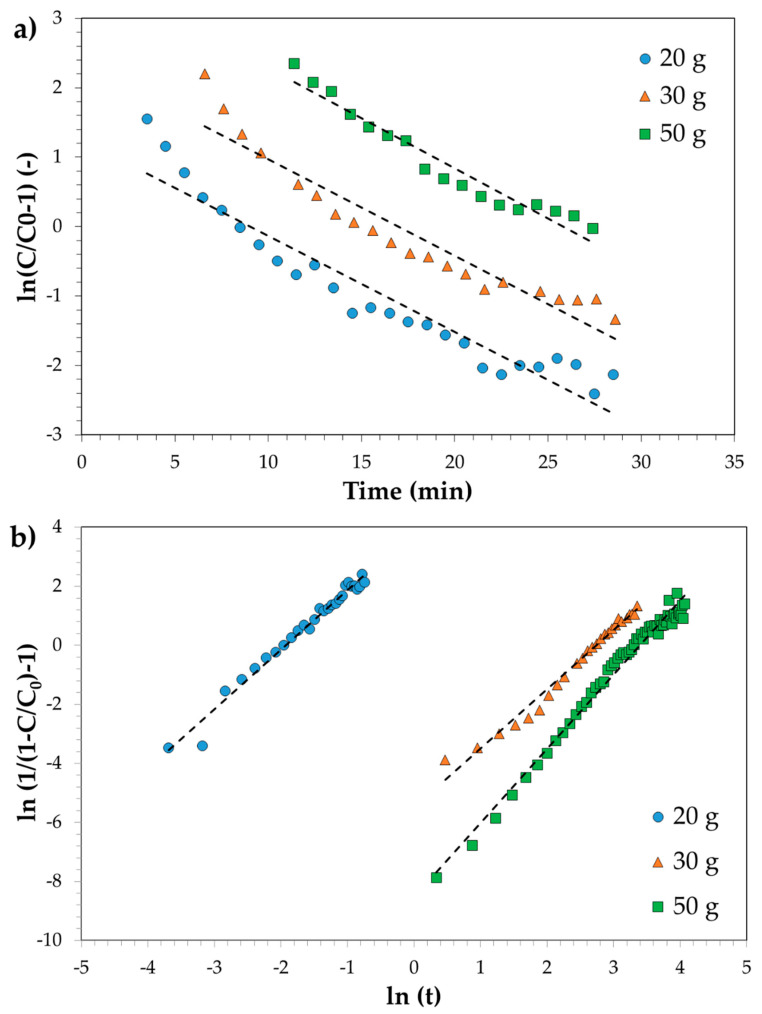
Linear plot of the Thomas (**a**) and MDR (**b**) models. Dashed lines refer to the fitted linear regression model.

**Figure 11:**
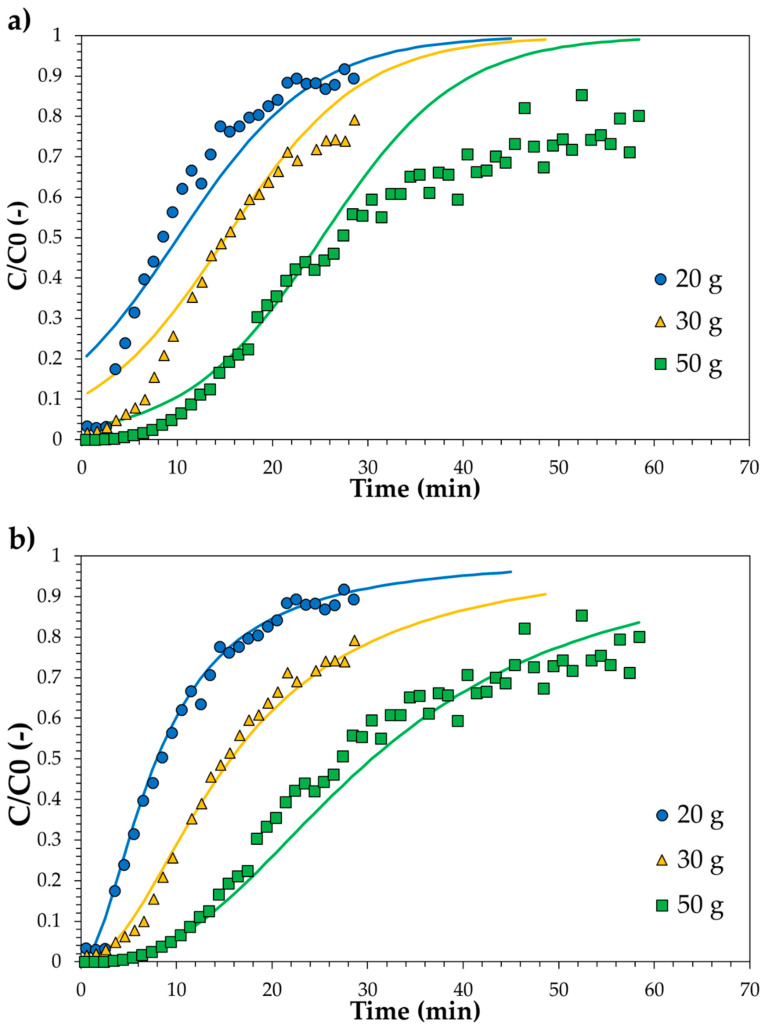
Comparison with experimental data of the Thomas (**a**) and MDR (**b**) models. Continuous lines refer to the fitted linear regression models.

**Figure 12:**
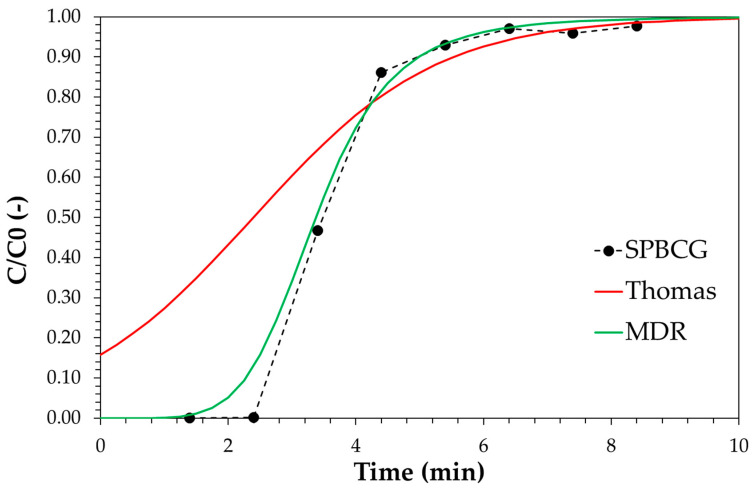
Comparison with experimental data of the Thomas (red line) and MDR (green line) models for the biochar SPBCG sample.

Figure 10 shows the linear plot of the investigated models, and the estimated parameters are reported in Table 3. The comparison between models and experimental data is reported in Figure 11. Results clearly show that the MDR model satisfactorily fits the experimental data in the whole time range and for each carbon amount. On the contrary, the Thomas model diverges from experimental data at low time values and for higher carbon amounts. It may be then concluded that MDR is better than the Thomas model. Both Thomas and MDR models were also applied for describing the breakthrough curve of the SPBCG sample and results are reported in Figure 12. The model parameters estimated by linear regression are reported in Table 3. As in the case of SP1000, the MDR model may be considered the best fitting mathematical model of the breakthrough curve of the SPBCG sample.

**Figure 13:**
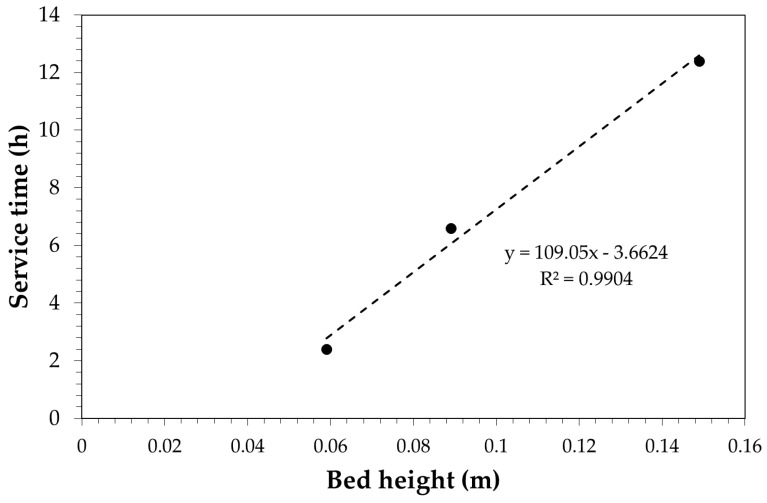
Linear plot of the BDST model. In the linear regression model, y refers to the service time (h) and x refers to the bed height (m).

On the basis of the obtained breakthrough curves, scale-up may be carried out by the Bed Depth Service Time (BDST) model, which may be expressed as follows:

(3) $$t=\frac{N\cdot H}{C_{0}\cdot v}-\frac{1}{k\cdot C_{0}}ln\left(\frac{C_{0}}{C_{b}}-1\right)$$

where *C*_0_ is the initial concentration of phenol, *C_b_* is the breakpoint concentration, *v* is the liquid empty bed velocity, *t* is the service time, *H* is the bed height, *N* is the adsorption capacity of the bed and *k* is a kinetic parameter. By plotting the service time obtained experimentally vs. bed height it is possible to obtain N and k values, which are the scale-up parameters. Figure 13 shows the plot of the BDST equation applied to the obtained experimental data.

The value of the BDST model are N = 45,438 g/m^3^ and k = 7.2 × 10^−3^ m^3^/h/g. Therefore, the obtained parameters may be used to estimate the service time as a function of bed height, superficial velocity, and desired breakpoint concentration.

Another practical tool to estimate the service time is to consider a design adsorption capacity (*W_design_*) as 50% lower than that calculated from the isotherm (*W_theoretical_*) [57]:

(4) *W_design_* = 0.5 *W_theoretical_*

On the basis of obtained results, Langmuir isotherms may be used to calculate W_theoretical_ as a function of initial phenol concentration. The carbon usage rate (CUR) may be then estimated as follows:

(5) $$CUR=\frac{Q\cdot C_{0}\cdot \left(1-\frac{C_{b}}{C_{0}}\right)}{W_{design}}$$

where *Q* is the wastewater flowrate (m^3^/h), *C*_0_ is the phenol concentration in the wastewater (g/m^3^), *C_b_* is the maximum admissible concentration in the treated stream, i.e., breakpoint concentration, and *W_design_* is the design adsorption capacity (g_PhOH_/kg_carbon_). As an example, Table 4 shows the CUR (kg_carbon_/h) of the investigated samples estimated for treating 1 m^3^/h of wastewater contaminated with phenol for several initial concentrations.

By comparing the results, performance ratios ranging from a minimum value of about 3 (SPBCP vs. SP800) to a maximum value of around 40 (SPBCP vs. SP1000) can be estimated, depending on the specific materials and the concentration of phenol in the solution. Concerning the two biochars, no significant differences may be observed for low phenol concentration values, while a lower carbon usage rate is calculated for pyrolysis biochar with respect to gasification biochar at high phenol concentration.

**Figure 14:**
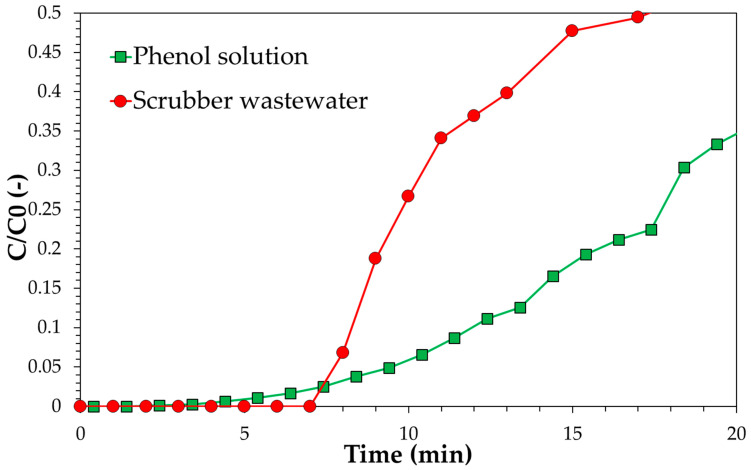
Breakthrough curve of the SP1000 sample for the phenol model solution and real scrubber wastewater. Solution initial phenol concentration: 5000 mg/g, flowrate: 55 mL/min, carbon amount: 50 g.

A continuous test was also carried out for real scrubber wastewater over commercial activated carbon in order to obtain insights about the suitability of phenol as a model molecule for wastewater purification. Results are reported in Figure 14.

Results of continuous tests confirm data obtained during batch adsorption tests. The level of stream purification is lower in the case of real scrubber wastewater. In particular, if *C*/*C*_0_ = 0.1 is assumed as the breakpoint value, the service time is reduced from 12 min to about 8 min. This may be also considered as a design parameter. Therefore, based on the collected data, the CUR estimated for phenol should be increased by a factor 12/8 = 1.5 in the case of a real scrubber wastewater stream. GC-MS analysis was carried out on both fresh and treated wastewater in order to assess the effect of other molecules on phenol removal. Wastewater consists of phenol as the main hydrophilic tar, with the presence of indene, a hydrophobic tar. The presence of indene may be responsible for the lower purification level observed in the case of real wastewater, especially for materials with low adsorption capacity. Indeed, in the case of the SPBCG sample, after 24 h treatment under batch conditions with 4 g of carbon, indene was completely removed, while phenol was still found in the residual solution, indicating that the adsorption of hydrophobic tars, e.g., indene hinders the adsorption of hydrophilic tars, e.g., phenol. Therefore, in the case of a syngas cleaning system consisting of a water scrubber downstream of a biodiesel scrubber, the latter unit should be suitably designed in order to avoid the presence of hydrophobic tars in the syngas, with a negative effect on the purification of wastewater produced in the secondary scrubber.

The obtained results may be exploited for both technical and economic assessments. For instance, the utilisation of biochar produced from a gasification well agrees with the concept of the circular economy. In fact, the biochar produced during gasification is considered a residual stream usually treated as a waste. Therefore, the estimated carbon usage rate gives information about the possibility to reuse the produced biochar within the same gasification plant before its disposal. In that scenario, it is important to note that, in the case of a biomass gasification plant fuelled with commonly used lignocellulose feedstocks such as poplar wood, almond shell, pine wood, eucalyptus, wheat straw, poultry litter, maize cobs, grape marc, miscanthus, and switchgrass, the biochar yield is usually lower than 20% with respect to the initial biomass flowrate to the gasifier [58]. Furthermore, both the type of gasifier and the operation conditions also strongly affect the formation of tars, with an effect on the amount and the characteristics of wastewater to be treated; this aspect should also be taken into account for the assessment. For instance, tar content in the syngas may be up to 100–150 g/Nm^3^ in the case of an updraft gasifier, and lower than 10 g/Nm^3^ in the case of a downdraft gasifier, and phenolic compounds represent usually about 10% of the total tar content [59,60,61,62]. Future research should focus on technical and economic assessment of the possible utilization of biochar as a partial or complete substitute to commercial activated carbons for the purification of wastewater contaminated with phenols at pilot or demonstrative scale.

## 4. Conclusions

In this work, two commercial activated carbons and two residual biochars obtained from pyrolysis and gasification processes were assessed as potential adsorbents for the purification of wastewater produced in a syngas wet scrubber unit of a biomass gasification plant of concern at the ENEA Trisaia Research Centre. Phenol solution was used as a model solution for the investigations. Data obtained from both batch and continuous tests were used to compare the investigated samples in terms of kinetics and adsorption capacity. On the basis of results obtained at bench-scale, design parameters were also estimated. Experimental tests indicate that the phenol adsorption capacity estimated by the Langmuir model depend on the pore volume of the carbons. Continuous tests give evidence of the lower efficacy of the considered residual biochars, compared to the commercial activated carbons. Design parameters were estimated by applying the BDST model. Furthermore, a practical tool was also used for estimating the carbon usage rate (CUR) and a case study was assessed. Based on the collected results, the two investigated biochars exhibit a similar CUR at low phenol concentration, while a lower CUR is estimated for pyrolysis biochar with respect to gasification biochar at a high phenol concentration level.

Overall, the collected results indicate that the performance of the biochars are significantly lower than commercial materials; nevertheless, the residual chars under examination have proven a certain degree of effectiveness as activated carbons that can be used for the regeneration of water contaminated by phenols. This achievement therefore provides an incentive for further evaluation, both technical and economic, with respect to their use for the regeneration of water contaminated by phenols and the design of a related plant unit.

## Figures and Tables

**Table 1:** B.E.T. specific surface area and pore volume of the investigated samples.

Sample	B.E.T. SSA (m^2^/g)	B.E.T C-Value (−)	Total Pore Volume (cm^3^/g)
SP1000	1281	6289	0.643
SP800	698	6155	0.413
SPBCP	211	81	0.126
SPBCG	63	844	0.069

**Table 2:** Parameters estimated from the breakthrough curve of SP1000 for phenol solution.

Carbon Amount (g)	Bed Height (cm)	Total Adsorption Capacity (mg/g)	Breakthrough Time ^a^ (min)	Breakthrough Adsorption Capacity ^b^ (mg/g)	Stoichiometric Time ^c^ (min)
20	5.9	133	3	40	11
30	8.9	152	6	49	21
50	14.9	177	12	71	37

^a^ Calculated at C/C0 = 0.1; ^b^ adsorption capacity at C/C0 = 0.1; ^c^ calculated in accordance with [56].

**Table 3:** Parameters estimation of the Thomas and MDR models for the SP1000 and SPBCG samples.

Sample	Carbon Amount (g)	Thomas			MDR		
		Kth × 10^2^ (min/mL/mg)	q_th_ (mg/g)	R^2^	a (−)	q_MDR_ (mg/g)	R^2^
SP1000	20	2.76	118	0.959	1.8	109	0.998
	30	2.78	155	0.987	2.0	144	0.997
	50	2.88	142	0.985	2.5	168	0.984
SPBCG	50	14.01	13.1	0.949	5.6	19.5	0.995

**Table 4:** Carbon usage rate (CUR) for the SP1000, SP800, SPBCG and SPNCP samples as a function of phenol concentration. Wastewater flowrate: 1 m^3^/h.

CUR (kg/h)	SP1000	SP800	SPBCG	SPBCP
C0 = 5000 ppm	41	46	187	132
C0 = 2500 ppm	21	24	110	84
C0 = 1000 ppm	8	11	64	55
C0 = 500 ppm	4	7	48	45
C0 = 100 ppm	1	4	36	38

## Data Availability

All data are reported in the paper.
